# I_CAN_ (TRPM4) Contributes to the Intrinsic Excitability of Prefrontal Cortex Layer 2/3 Pyramidal Neurons

**DOI:** 10.3390/ijms22105268

**Published:** 2021-05-17

**Authors:** Denise Riquelme, Francisco A. Peralta, Franco D. Navarro, Claudio Moreno, Elias Leiva-Salcedo

**Affiliations:** Department of Biology, Faculty of Chemistry and Biology, Universidad de Santiago de Chile, Santiago 9170002, Chile; denise.riquelme@usach.cl (D.R.); francisco.peralta.p@usach.cl (F.A.P.); franco.navarro@usach.cl (F.D.N.); claudio.moreno@usach.cl (C.M.)

**Keywords:** TRPM4, afterdepolarization, medial prefrontal cortex, layer 2/3, intrinsic excitability

## Abstract

Pyramidal neurons in the medial prefrontal cortical layer 2/3 are an essential contributor to the cellular basis of working memory; thus, changes in their intrinsic excitability critically affect medial prefrontal cortex (mPFC) functional properties. Transient Receptor Potential Melastatin 4 (TRPM4), a calcium-activated nonselective cation channel (CAN), regulates the membrane potential in a calcium-dependent manner. In this study, we uncovered the role of TRPM4 in regulating the intrinsic excitability plasticity of pyramidal neurons in the mouse mPFC layer of 2/3 using a combination of conventional and nystatin perforated whole-cell recordings. Interestingly, we found that TRPM4 is open at resting membrane potential, and its inhibition increases input resistance and hyperpolarizes membrane potential. After high-frequency stimulation, pyramidal neurons increase a calcium-activated non-selective cation current, increase the action potential firing, and the amplitude of the afterdepolarization, these effects depend on intracellular calcium. Furthermore, pharmacological inhibition or genetic silencing of TRPM4 reduces the firing rate and the afterdepolarization after high frequency stimulation. Together, these results show that TRPM4 plays a significant role in the excitability of mPFC layer 2/3 pyramidal neurons by modulating neuronal excitability in a calcium-dependent manner.

## 1. Introduction

Calcium-activated nonselective cation (CAN) currents are activated by increases in intracellular calcium (Ca^2+^_i_) from internal reservoirs through G_q_-protein coupled receptors, ionotropic receptors [1,2,3], voltage gated calcium channels (VGCC) [4,5] or other sources of Ca^2+^ [6,7]. The Transient Receptor Potential Melastatin 4 (TRPM4), is a CAN channel highly sensitive to Ca^2+^_i_ [8,9] and permeable only to monovalent cations. TRPM4 is widely expressed in brain areas including the medial prefrontal cortex (mPFC) [10,11], hippocampus [12], accessory olfactory bulb [13], supraoptic nucleus [14], and cerebellum [15]. In these areas, TRPM4 plays roles in neuronal firing and synaptic plasticity.

In most of the areas where it is expressed, TRPM4 has been described in mechanisms regulating neuronal firing. For instance, in neurons of the accessory olfactory bulb [13], preBötzinger neurons [16,17], and in dopaminergic neurons of the *substantia nigra pars compacta* [18], TRPM4 participates in the plateau potential, increasing neuronal firing. In CA1 pyramidal neurons, TRPM4 activation increases postsynaptic depolarization, allowing the full activation of NMDA receptors [12]. In cerebellar Purkinje neurons, TRPM4 is responsible for the slow depolarizing current, which is essential for the activation of an afterdepolarization (ADP) [15]. In the entorhinal cortex, TRPM4 inhibition reduces burst firing [3], and in layer 5 mPFC pyramidal neurons, TRPM4 appears to have a minor role in the activation of the slow ADP [10] responsible for the transient fast firing observed in these neurons [19,20]. Finally, in layer 2/3 mPFC pyramidal neurons, a TRPM4-like current is active at the resting membrane potential [11].

Layer 2/3 pyramidal neurons are the most abundant cells in the neocortex [21], and they form excitatory inter-and intralaminar synapses. These projections control subcortical areas through indirect connections via deep layer neurons, which then project to areas such as the striatum, thalamus, and pontine nuclei [22,23]. Layer 2/3 pyramidal neurons receive excitatory inputs from subcortical areas such as the thalamus via layer 1 [24], deep layers in the cortex (5 and 6), and through neurons in the same layer 2/3 forming local circuits. Layer 2/3 pyramidal neurons in the prefrontal cortex are highly resistant to noise disturbance and participate in delayed activity during memory tasks, an important mechanism for working memory and mnemonic processing [25,26,27]. These neurons display backpropagating action potentials with an associated Ca^2+^ influx, and they can sustain burst firing associated with the activation of ADP [28]. Despite the abundance of TRPM4 in these neurons, the role of the channel in this area is still poorly understood.

Synaptic potentiation persistently changes the intrinsic excitability of the neuron. It is produced by changes in the expression/location or modification of the biophysical properties of the ion channels present in the membrane [29,30]. In these processes, Ca^2+^_i_ participates in the setting of the excitability levels by modulating the activity of Ca^2+^-dependent conductances such as K^+^, Cl^−^, and nonselective cationic currents (I_CAN_), which in turn modify synaptic integration, firing frequency, and firing patterns [19,31,32], all of which are critical for information processing and transmission; thus, the expression of I_CAN_ TRPM4 in layer 2/3 mPFC pyramidal neurons and its Ca^2+^_i_ dependency make this channel an attractive candidate for the regulation of intrinsic excitability.

Here, we investigate the participation of TRPM4 in the regulation of the intrinsic excitability of pyramidal neurons in the mPFC layer 2/3. Furthermore, we studied the role of TRPM4 before and after synaptic stimulation. We found that after synaptic stimulation, I_CAN_ TRPM4 increases the intrinsic excitability through the activation of a Ca^2+^_i_-dependent ADP; in contrast, in non-synaptically stimulated neurons, TRPM4 modulates the resting membrane potential.

## 2. Results

### 2.1. TRPM4 Participation in Pyramidal Neuron Spiking

**Figure 1:**
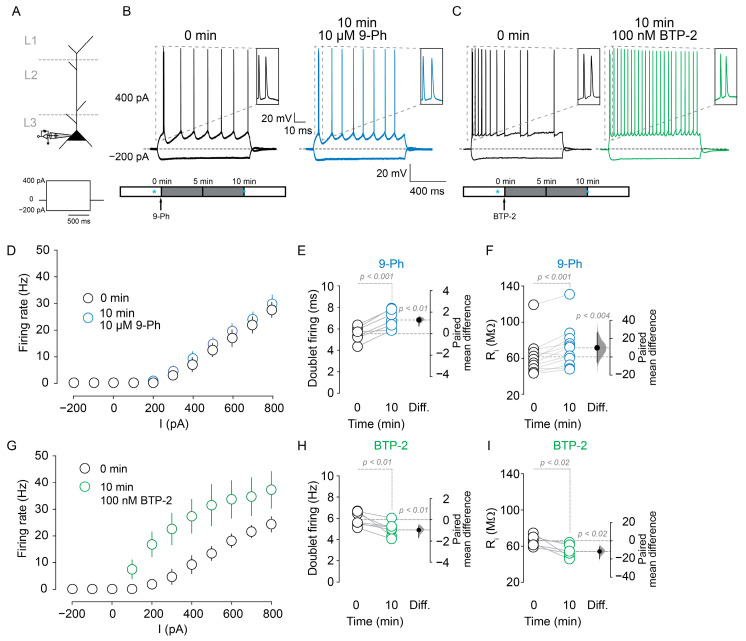
Effect of TRPM4 pharmacological inhibition on the firing properties of pyramidal neurons at mPFC layer 2/3. (**A**) Shows the recording configuration and experimental protocol. (**B**) Representative perforated patch voltage traces recorded in pyramidal neurons showing the effect of 10 µM 9-Ph. Bottom panel shows the experimental protocol, blue * represent the time where measures were taken; (**C**) shows the effect of 100 nM BTP-2, the voltage response evoked by −200 and 400 pA are shown, and the insets show the first two action potentials at the beginning of the current step. Bottom panel shows the experimental protocol, blue * represent the time where measures were taken. (**D**) Graph showing the frequency of action potential firing evoked by increasing somatic current injection (−200 to 800 pA, steps of 100 pA) in the control condition (black) and after 10 µM 9-Ph treatment (blue). (**E**) Shows the quantification of the time between the first two action potentials at the beginning of the depolarizing current pulse before and after 10 µM 9-Ph. (**F**) Shows the input resistance measured using a −50 pA, 50 ms pulse, 200 ms after the end of each current step. (**G**) Graph showing the frequency of action potential firing evoked by increasing somatic current injection (−200 to 800 pA, steps of 100 pA) in the control condition (black) and after 100 nM BTP-2 treatment (green). (**H**) Shows the quantification of the time between the first two action potentials at the beginning of the depolarizing current pulse before and after 100 nM BTP-2. (**I**) Shows the input resistance measured using a −50 pA, 50 ms pulse, 200 ms after the end of each current step. On the right side of each plot, the paired mean difference between the treatment conditions is shown; the mean difference is depicted as a dot; the 95% confidence interval is indicated by the end of the vertical error bar. Statistical differences were evaluated using a two-side permutation *t*-test and *p* values are shown above the dotted grey line.

To investigate the participation of TRPM4 in the intrinsic excitability of pyramidal neurons in layer 2/3 of the mPFC, we performed nystatin perforated patch-clamp recordings to minimize the experiment-induced changes in Ca^2+^_i_ buffering. First, we tested the effect of TRPM4 pharmacological inhibition on the intrinsic excitability. We injected a series of depolarizing current steps into the soma (−200 to 800 pA) to evoke neuron spiking (Figure 1A). Neurons treated with 10 µM 9-Phenathrol (9-Ph, a TRPM4 inhibitor) (Figure 1A, bottom panel) show similar firing frequency as the control condition (Figure 1B,C). Conversely, we measured the doublet firing as an indicator of the fast afterdepolarization [33] and observed that 9-Ph increases the time between the firings of doublets at the beginning of the step (0 min = 5.5 ± 0.7 ms; 10 min 9-Ph = 6.9 ± 0.8 ms, *p* < 0.001, paired *t*-test, *n* = 7; Figure 1B inset and Figure 1E). Additionally, after 10 µM 9-Ph application, we found an increase in the input resistance (R_i_) (0 min = 61.8 ± 20.2 MΩ, 10 min 9-Ph = 71.6 ± 22.6 MΩ, *p* < 0.001, paired *t*-test, *n* = 12; Figure 1F).

**Figure 2:**
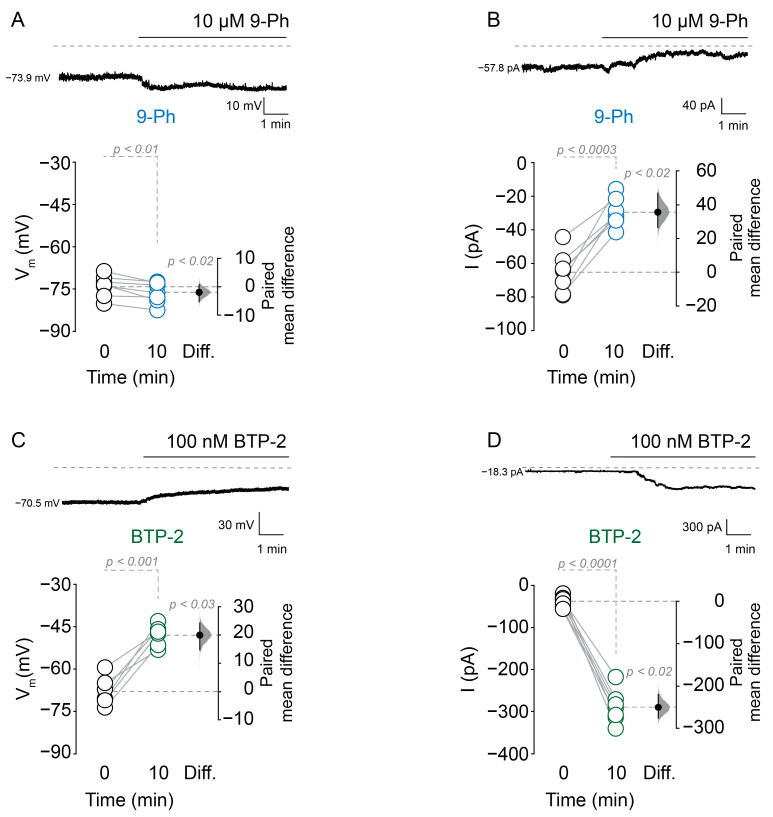
TRPM4 inhibition hyperpolarizes the resting membrane potential. Representative perforated patch traces of membrane potential (**A**) and current (**B**) (V_h_ = −70 mV) before and during 10 µM 9-Ph application. Bottom panels show the summary plots of membrane potential and current changes induced by 9-Ph application. Representative perforated patch traces of membrane potential (**C**) and current (**D**) before and during 100 nM BTP-2 application. Bottom panels show the summary plots of the effect of 100 nM BTP-2. On the right side of each plot, the paired mean difference between conditions is shown; the mean difference is depicted as a dot; the 95% confidence interval is indicated by the end of the vertical error bar. Statistical differences were evaluated using a two-side permutation *t*-test and *p* values are shown above the dotted grey line.

Next, we sought to determine the effect of TRPM4 inhibition on the resting membrane potential. Using perforated patch-clamp recordings, we found the application of 10 µM 9-Ph hyperpolarized the membrane potential (0 min = −73.9 ± 3.9 mV, 10 min 9-Ph = −76.5 ± 3.4 mV, *p* < 0.01, paired *t*-test, *n* = 7; Figure 2A) and decreased the current (0 min = −65.1 ± 12.1 pA, 10 min 9-Ph = −28.9 ± 8.6 pA; holding potential (V_h_) = −70 mV; *p* < 0.0003, paired *t*-test, *n* = 7; Figure 2B); conversely, the bath application of 100 nM BTP-2 (3,5-bis(trifluoromethyl)pyrazole, a TRPM4 open channel potentiator) depolarized the membrane potential (0 min = −67.8 ± 5.2 mV, 10 min BTP-2 = −47.8 ± 3.8 mV, *p* < 0.001, paired *t*-test, *n* = 6; Figure 2C) and increased the current (0 min = −37.4 ± 13.1 pA, 10 min BTP-2 = −290.1 ± 39.1 pA, V_h_ = −70 mV, *p* < 0.0001, paired *t*-test, *n* = 7; Figure 2D). Together, these results indicate that a fraction of TRPM4 is active at resting membrane potential in these neurons, which is consistent with the reported Ca^2+^_i_ affinity of TRPM4 [8] and our experiments in overexpression systems (Supplementary Figure S1).

### 2.2. TRPM4 Inhibition Reduces Action Potential Firing after High Frequency Stimulation

**Figure 3:**
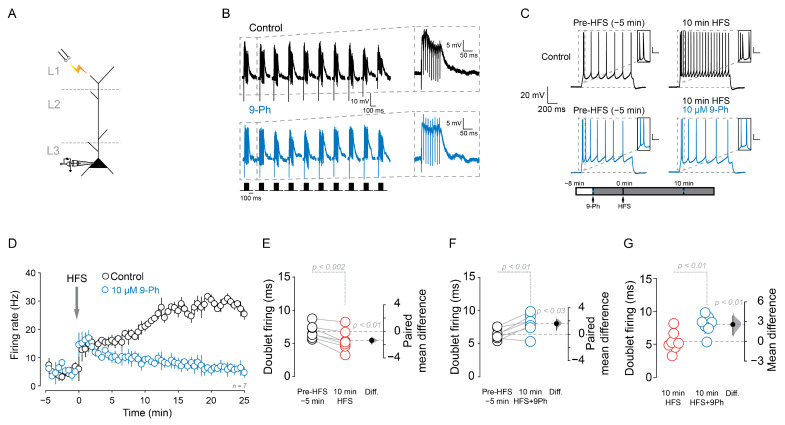
TRPM4 pharmacological inhibition reduces the firing frequency induced by high frequency stimulation. (**A**) Scheme of the recording and synaptic stimulation configuration. (**B**) Shows the representative perforated patch voltage traces during HFS and the effect of 9-Ph (blue trace), below it is the HFS protocol. (**C**) Representative perforated patch current-clamp recording showing pre-HFS, 10 min after HFS, pre-HFS + 9-Ph, and 10 min after HFS + 9-Ph; insets show the doublet firing at the beginning of the current pulse. Bottom panel shows the experimental protocol, blue * represent the time where measures were taken (**D**) Graph showing the temporal change in the firing frequency after HFS and HFS + 9-Ph treatment. (**E**–**G**) show the quantification of the time between the first two action potentials evoked by current injection before and after HFS (**E**), HFS + 9-Ph (**F**) and the comparison between HFS and HFS + 9-Ph at 10 min (**G**). On the right side of each plot the paired mean difference (**E**,**F**) and the mean difference (**G**) between treatment conditions is shown; the mean difference is depicted as a dot; the 95% confidence interval is indicated by the end of the vertical error bar. Statistical differences were evaluated using a two-side permutation *t*-test and *p* values are shown above the dotted grey line. Calibration bar in the insets corresponds to 20 mV and 10 ms.

Next, we evoked excitatory postsynaptic potentiation by delivering a high frequency stimulation (HFS) in layer 1 and recorded from layer 2/3 pyramidal neurons in the mPFC using nystatin perforated patch clamp configuration (Figure 3A). We found that HFS of the layer 1 (Figure 3B, see methods) resulted in the increase in AP firing frequency after a somatic current injection above the rheobase (400 pA) (pre-HFS = 7.4 ± 2.5 Hz; 10 min after HFS = 20.9 ± 2 Hz, *n* = 7, *p* < 0.02, paired *t*-test; Figure 3C,D) and reducing the time between the initial doublets APs (pre-HFS = 6.8 ± 1.1 ms; 10 min HFS = 5.4 ± 1.6 ms, *p* < 0.002, paired *t*-test, *n* = 7; Figure 3C inset and Figure 3E). The application of 10 µM 9-Ph prevented the prolonged firing frequency increase (10 min-HFS = 20.9 ± 2 Hz; 10 min after HFS + 9-Ph = 9.5 ± 1.9 Hz, *n* = 7, *p* < 0.001, unpaired *t*-test, Figure 3C,D) and increased the time between the initial doublet firing after HFS (pre-HFS = 6.4 ± 0.8 ms; 10 min after HFS + 9-Ph = 8.01 ± 1.4 ms, *p* < 0.01, paired *t*-test, *n* = 7; Figure 3C inset and Figure 3F). Additionally, a comparison between 10 min after HFS and 10 min after HFS + 9-Ph showed an increase in the time between the initial doublet firing (*p* < 0.01, unpaired *t*-test, Figure 3G).

**Figure 4:**
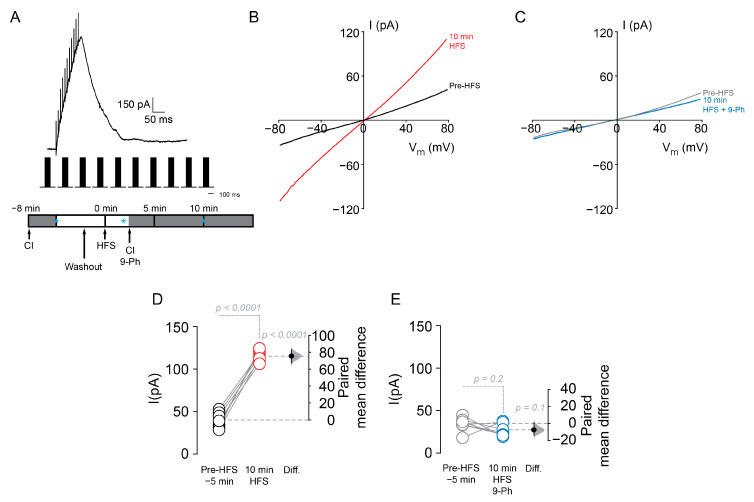
TRPM4 pharmacological inhibition reduces the CAN current activated by high frequency stimulation. (**A**) Shows the representative perforated patch current trace during HFS, below it is the HFS protocol and the experimental design showing the time when the drug was applied, blue * indicate the time where the currents were taken for the (**D**,**E**) plots. (**B**) Representative perforated patch current traces pre-HFS (black) and 10 min after HFS (red). (**C**) Representative perforated patch current traces pre-HFS (grey), and 10 min HFS + 10 µM 9-Ph (blue). Summary plot showing the current measured before and 10 min after the HFS (**D**) and in the presence of 9-Ph (**E**). On the right side of each plot, the paired mean difference between treatment conditions is shown; the mean difference is depicted as a dot; the 95% confidence interval is indicated by the end of the vertical error bar. Statistical differences were evaluated using a two-side permutation *t*-test and *p* values are shown above the dotted grey line.

To determine whether the effect of HFS on excitability is linked to the activation of a TRPM4-like current, we isolated the current by including a cocktail of inhibitors (DL-AP5 to inhibit NMDA currents, CNQX to inhibit AMPA receptors, TTx to inhibit voltage-dependent Na^+^ currents, CdCl_2_ to inhibit voltage-dependent Ca^2+^ currents, and Picrotoxin to inhibit GABAA currents, CI, see Methods) in the extracellular buffer, a Cs-based intracellular solution (see Methods) and measured the remaining current using a voltage ramp (−80 to 80 mV, 0.32 mV/ms, V_h_ = −70 mV) in the perforated patch clamp configuration. We then washed out CI and delivered the HFS protocol while holding the recorded neuron at 0 mV, and after that, we included CI and measure the remaining currents (Figure 4A, bottom panel). We found that HFS increased a 9-Ph sensitive current (pre-HFS = 39.4 ± 9.2 pA; 10 min after HFS = 115.6 ± 6.4 pA; *n* = 7, paired *t*-test, *p* < 0.0001, Figure 4B,D; pre-HFS = 33.6 ± 8.2 pA; 10 min after HFS + 10 µM 9-Ph = 26.3 ± 7.4 pA; *n* = 7, paired *t*-test, *p* = 0.2, Figure 4C,E) with a reversal potential close to 0 mV (Figure 4B,C), suggesting that this current could be involved in the increase in the firing frequency induced by HFS.

### 2.3. TRPM4 Silencing Reduces the Action Potential Firing after High Frequency Stimulation

**Figure 5:**
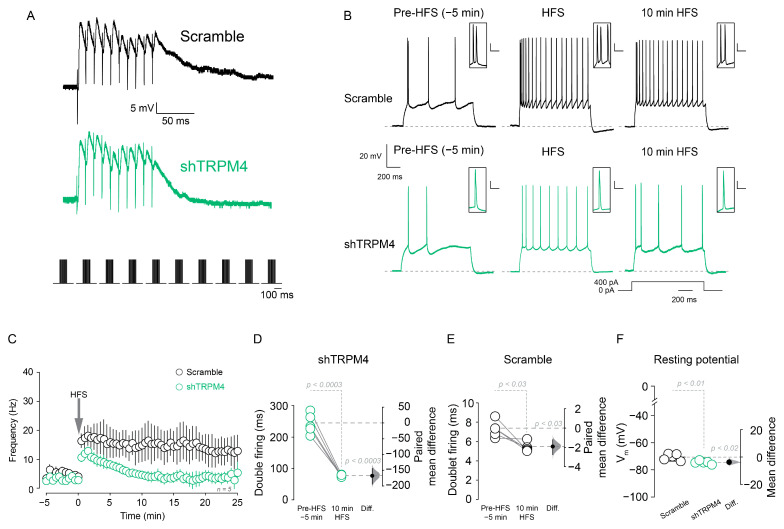
TRPM4 silencing reduces the action potential firing after HFS. (**A**) Shows the representative voltage response during HFS in scramble (black trace) and shTRPM4 (green trace) expressing neurons (only the first pulse) recorded using perforated patch configuration, below it is the HFS protocol. (**B**) Representative perforated patch current-clamp recordings showing pre-HFS, 2 min, and 10 min post-HFS in scramble (black traces) and shTRPM4 (green traces) expressing neurons, insets showing doublet firing. (**C**) Graph showing the temporal change in the firing frequency in both scramble and shTRPM4 expressing neurons. (**D**) Shows the quantification of the time between the first two action potentials evoked by current injection before and after HFS in shTRPM4 expressing neurons and (**E**) in scramble expressing neurons. (**F**) Summary plot showing the resting membrane potential of scramble and shTRPM4 expressing neurons. The paired mean difference (**D**,**E**) and the mean difference (**F**) between pre-HFS and 10 min HFS is shown in the Gardner–Altman estimation plot. The mean difference is depicted as a dot; the 95% confidence interval is indicated by the ends of the vertical error bar. Statistical differences were evaluated using a two-side permutation *t*-test and *p* values are shown above the dotted grey line. Calibration bar in the insets corresponds to 20 mV and 10 ms.

To further confirm the role of TRPM4 in the increase in excitability, we used a shRNA mediated knock-down of TRPM4 (shTRPM4, Supplementary Figure S2). In shTRPM4 expressing neurons, we measured the firing frequency in response to 400 pA somatic current injection using the perforated patch clamp configuration. We found that the HFS-induced increase in firing frequency was lower in shTRPM4 than in the scramble (Figure 5B,C), the firing frequency drops steadily (Figure 5C) with an increase in the time of the doublet firing (pre-HFS = 243.3 ± 31.7 ms; 10 min after HFS = 75.7 ± 3.3 ms, *p* < 0.0003, paired *t*-test, *n* = 5; Figure 5B inset and Figure 5D). Conversely, the neurons transduced with scramble showed similar values as non-transduced neurons (pre-HFS = 7.3 ± 0.9 ms; 10 min after HFS = 5.5 ± 0.5 ms, *p* < 0.03, paired *t*-test, *n* = 5; Figure 5B inset and Figure 5E). Additionally, we measured the resting membrane potential of both scramble and shTRPM4 transduced neurons and found that shTRPM4 expressing neurons are hyperpolarized compared to scramble (scramble = −70.6 ± 2.5 mV, shTRPM4 = −74.6 ± 1.2 mV, *n* = 5, *p* < 0.01, unpaired *t*-test, Figure 5F).

**Figure 6:**
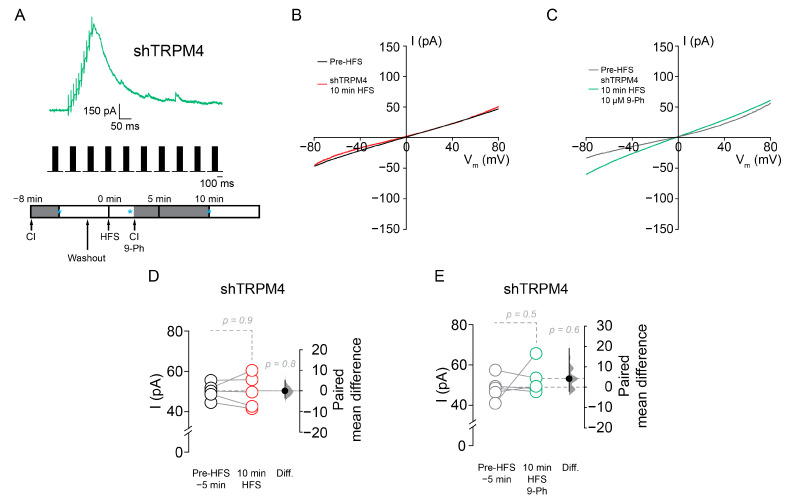
TRPM4 silencing reduces HFS induced CAN current. (**A**) Representative current responses during HFS recorded using perforated patch configuration, below it is the HFS protocol and the experimental design showing the time when the drug was applied, blue * indicate the time where the currents were taken for the analysis. (**B**) Representative current traces in shTRPM4 pre-HFS (black), and shTRPM4 10 min after HFS (red). (**C**) Representative current traces in and shTRPM4 pre-HFS (grey), and shTRPM4 10 min after HFS + 10 µM 9-Ph (green). Summary plot showing the current measured in shTRPM4 before and 10 min after the HFS (**D**) and in the presence of 9-Ph (**E**). On the right side of each plot the paired mean difference between treatment conditions is shown; the mean difference is depicted as a dot; the 95% confidence interval is indicated by the end of the vertical error bar. Statistical differences were evaluated using a two-side permutation *t*-test and *p* values are shown above the dotted grey line. Calibration bar in the insets corresponds to 20 mV and 10 ms.

Moreover, in shTRPM4 transduced neurons, HFS (Figure 6A) did not activate the CAN current (pre-HFS = 50.3 ± 4.1, 10 min after HFS = 50.2 ± 8.2 pA, *p* = 0.9 paired *t*-test, *n* = 5; Figure 6B,D), neither 9-Ph reduce the current (pre-HFS = 48.7 ± 5.9, 10 min after HFS = 52.9 ± 7.7 pA, *p* = 0.5, paired *t*-test; *n* = 5; Figure 6C,E). Together, these results suggest that TRPM4 activity takes part in the increase in the intrinsic excitability after synaptic stimulation.

### 2.4. TRPM4-Dependent Increase in ADP after HFS

**Figure 7:**
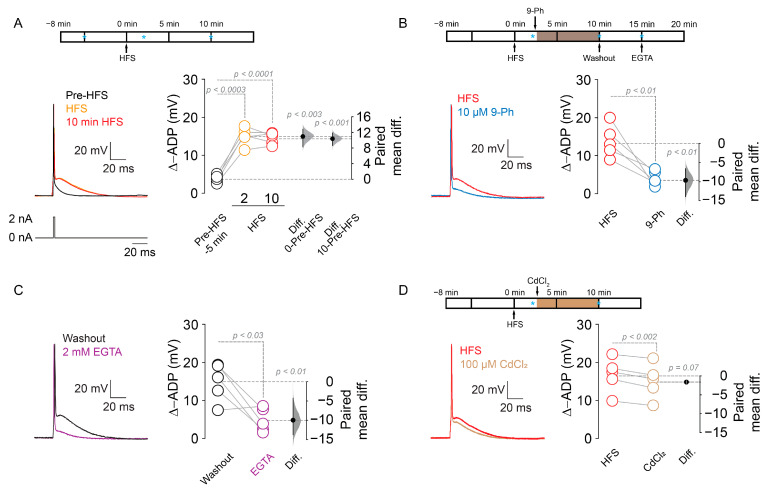
TRPM4 inhibition reduces ADP amplitude in HFS induced neurons. (**A**) Representative current-clamp experiments in perforated patch configuration showing the effect of HFS on the ADP; pre-HFS (black), immediately after HFS (orange) and 10 min after HFS (red), above it is showing the experimental design indicating the time when the drug was applied, blue * indicate the time where the currents were taken for the analysis. (**B**) Shows the effect of 10 µM 9-Ph on ADP amplitude (blue); above it is showing the experimental design indicating the time when the drug was applied, blue * indicate the time where the voltage was taken for the analysis. (**C**) Shows the recovery of the amplitude of ADP after 5 min 9-Ph washout (black), and the effect of intracellular EGTA (2 mM) after whole cell break-in (purple) in the HFS induced ADP, the experimental design is shown in (**C**). (**D**) Shows the effect of 100 µM CdCl_2_ (brown) on the HFS induced ADP; above it is showing the experimental design indicating the time when the drug was applied, blue * indicate the time where the currents were taken for the analysis. On the right side of each plot, the paired mean difference between the treatment conditions is shown; the mean difference is depicted as a dot; the 95% confidence interval is indicated by the end of the vertical error bar. Statistical differences were evaluated using a two-side permutation *t*-test and *p* values are shown above the dotted grey line.

The effect of 9-Ph on the firing frequency after HFS and in the initial doublet firing strongly suggests an effect on ADP [34]; we tested the role of TRPM4 in ADP. We delivered an HFS in layer 1 of the mPFC and then we measured ADP amplitude following a single action potential induced by a short duration highly depolarizing somatic current injection (2 ms, 2 nA) in pyramidal neurons in layer 2/3 of the mPFC using the perforated patch clamp configuration. We found that HFS increased the ADP amplitude (pre-HFS = 3.8 ± 1.1 mV, 2 min after HFS = 15.1 ± 2.3 mV, 10 min after HFS = 14.6 ± 1.52 mV, *p* < 0.0003, *p* < 0.0001 respectively, one-way ANOVA, Dunnet post hoc, *n* = 5; Figure 7A). Furthermore, the application of 10 µM 9-Ph reversed the HFS-induced increase in ADP (2 min after HFS = 13.6 ± 4.2 mV, 10 min after HFS + 9-Ph = 3.9 ± 1.9 mV, *p* < 0.01, paired *t*-test, *n* = 5; Figure 7B). Next, we assessed the Ca^2+^_i_ dependency of this effect by washing out 9-Ph to restore the ADP amplitude, then rupturing the cell membrane to obtain whole-cell configuration, allowing the diffusion of the Ca^2+^ chelator EGTA (2 mM) into the neuron. Using this experimental protocol, we observed a decrease in the ADP amplitude (5 min after washout = 14.7 ± 4.9 mV, EGTA = 4.7 ± 3.1 mV; *p* < 0.03, paired *t*-test, *n* = 5; Figure 7C), demonstrating that the ADP in these neurons depends on Ca^2+^_i_. Next, to test the influence of voltage-dependent Ca^2+^ channels (VGCC), we performed the same protocol in the presence of 100 µM CdCl_2_, a broad spectrum VGCC inhibitor, and found a reduction in the ADP (2 min after HFS = 16.8 ± 4.5 mV, CdCl_2_ = 15.2 ± 4.6, *p* < 0.002, paired *t*-test, *n* = 5, Figure 7D). The effect size of Cd^2+^ is lower than 9-Ph and EGTA (9-Ph = 9.7, EGTA = 9.9, Cd^2+^ = 1.6, Figure 7), suggesting that the 9-Ph sensitive component plays a major role in the ADP.

**Figure 8:**
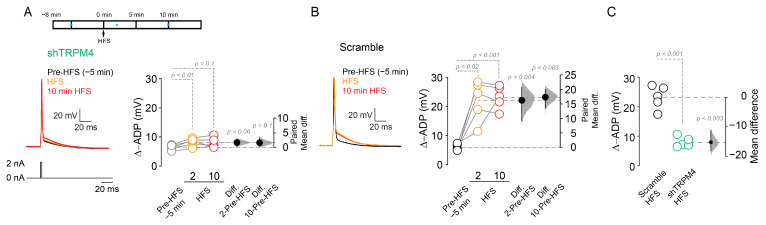
TRPM4 silencing reduces ADP amplitude in HFS induced neurons. (**A**) Shows the effect of HFS on ADP in shTRPM4 expressing neurons recorded in perforated patch configuration; above it is showing the experimental design; blue * indicate the time where the voltage was taken for the analysis. (**B**) Shows the effect of HFS on ADP in scramble expressing neurons. (**C**) Shows the comparison of the Δ-ADP between scramble and shRNA expressing neurons at 10 min after HFS. On the right side of each plot, the paired mean difference (**A**,**B**) and the mean difference (**C**) between treatment conditions is shown; the mean difference is depicted as a dot; the 95% confidence interval is indicated by the end of the vertical error bar. Statistical differences were evaluated using a two-side permutation *t*-test and *p* values are shown above the dotted grey line.

To confirm the participation of TRPM4 in the ADP, we recorded from shTRPM4 transduced neurons in the perforated patch configuration and found that HFS activates an ADP (pre-HFS = 6.3 ± 0.9 mV, 2 min after HFS = 8.1 ± 1.4 mV, 10 min after HFS = 8 ± 1.7 mV, *n* = 5; *p* < 0.01, *p* = 0.1 respectively, one-way ANOVA, Figure 8A,C) that is smaller than the ADP present in scramble transduced neurons (pre-HFS = 5.9 ± 1.1 mV, 2 min after HFS = 22.2 ± 7.1 mV, 10 min after HFS = 23.2 ± 3.9 mV, *n* = 5; *p* < 0.02, *p* < 0.001 respectively, one-way ANOVA, Figure 8B,C), also with an effect size at 10 min lower than the scramble (scramble = 17.3, shTRPM4 = 1.6, Figure 8). Together, these data shows that TRPM4 participates in the Ca^2+^-dependent ADP activation, thus modulating the firing frequency after HFS in the pyramidal neurons in layer 2/3 of the mPFC.

## 3. Discussion

In this study, we investigated the role of TRPM4 in regulating the intrinsic excitability of mPFC layer 2/3 pyramidal neurons. Our findings support the hypothesis that TRPM4 regulates the intrinsic excitability in resting conditions by taking part in the setting of the resting membrane potential and after synaptic stimulation by increasing the amplitude of the afterdepolarization mediated by Ca^2+^_i_. Together, our data offer an intriguing link between Ca^2+^, synaptic plasticity, and intrinsic excitability mediated by TRPM4; this mechanism could have important effects on the physiology of mPFC layer 2/3 pyramidal neurons.

### 3.1. TRPM4 Activity at Resting Membrane Potential

We found that, under resting conditions, a fraction of TRPM4 is active, consistent with its Ca^2+^ sensitivity (0.05–10 µM) and the resting Ca^2+^_i_ concentrations (0.05 to 0.2 µM [35]), which are enough to activate the channel [8,9]; these results suggest that TRPM4 contributes to the setting of the resting membrane potential of pyramidal neurons in the mPFC L2/3. Similarly, FFA (which also inhibits TRPM4) and Ca^2+^_i_ chelation hyperpolarized the accessory olfactory bulb neurons [13]. In this respect, most of the experiments measuring the membrane potential used conventional whole cell, and this may allow the diffusion of Ca^2+^ buffering proteins that could be altering the function of TRPM4 and possibly hiding some of the effects of TRPM4 [36,37,38,39].

Previous reports show that 9-Ph activates the Ca^2+^-activated K^+^ channel K_Ca_3.1 [40,41] and inhibits the Ca^2+^-activated Cl^-^ channel TMEM16A [31,42,43]. In this context, our results indicate that 9-Ph increases the input resistance and hyperpolarizes the membrane potential, contrary to the expected effect of 9-Ph on K_Ca_3.1 (decrease in input resistance) and TMEM16A (depolarization) [31]. Additionally, BTP-2 potentiates TRPM4 currents only when the channel is open, depolarizing the neurons [44]; however, at higher concentrations, BTP-2 inhibited Ca^2+^ release-activated Ca^2+^ current (I_CRAC_) (EC_50_ = 150 nM) with no effect on store-operated Ca^2+^ entry (SOCE) channels (EC_50_ = 4.7 µM) [45]. Although in our experiments, the concentration of BTP-2 (100 nM) is enough to inhibit I_CRAC_, this inhibition can only affect the Ca^2+^_i_ concentration and not the membrane potential [46,47].

### 3.2. TRPM4 Role in Intrinsic Excitability

TRPM4 is expressed in the apical proximal dendrite of pyramidal neurons in the L2/3 of the mPFC; this area receives inputs from L1, L2/3, and L4, but primarily from thalamocortical input from layer 1 [24,48]. The finding that 9-Ph increases the time between the first and the second AP (doublet firing) after synaptic stimulation suggests that TRPM4 has a different effect in the neuron depending on their activity state. How can this effect be related to the physiology of the neuron? In resting conditions, the activity of TRPM4 may take part in the setting of the resting membrane potential; however, after a train of action potentials, the activity of TRPM4 could be manifested by the increase in Ca^2+^ influx induced by the triggering of APs. In this context, T-type, R-type, and L-type Ca^2+^ channels [49] contribute to the shape of the AP through the modulation of Ca^2+^-activated channels such as SK [50,51] BK [52,53], and TMEM16B [31]; in this case, VGCC could be modulating I_CAN_ and neuronal firing. In this way, in thalamic reticular nucleus neurons, T-type and possible R-type channels provide the Ca^2+^ necessary for TRPM4 activation that contributes to the persistent firing; thus, it is plausible to hypothesize a similar mechanism in L2/3 pyramidal neurons in the mPFC [54].

Our data indicate that 9-Ph reduces the firing frequency and increases the time between duplex firing in HFS-stimulated neurons for a prolonged time. Moreover, TRPM4 silencing reduced the firing frequency and increased the time between doublet firing, indicating that the increase in the firing rate after HFS is partially dependent on the activity of TRPM4. Furthermore, the data showed that HFS increases the firing rate in TRPM4-silenced neurons in the first ~5 to 10 min, suggesting that other mechanisms such as persistent Na^+^ currents or VGCC activation could be sustaining this effect immediately after HFS, while TRPM4 keeps the activity at later times.

HFS unveiled a strong contribution of TRPM4 in the control of neuronal excitability in pyramidal neurons of the mPFC layer 2/3 and the observation that TRPM4 produced a prolonged increase in AP firing, suggesting a long-lasting modification of the channel function. In this respect, a change in TRPM4 localization or the relocalization of the Ca^2+^ source near TRPM4 (VGCC, RyR or IP_3_R) could explain such long-term effects in the AP firing. Additionally, TRPM4 presents several residues for PKC phosphorylation [55], making it probable that the increase in TRPM4 activity could be related to post-translational modification, directly changing the activity of the channel or by inducing the upregulation or relocalization of the channel in neurons in the proximity of the Ca^2+^ source.

### 3.3. TRPM4-Dependent ADP Activation Role in Intrinsic Excitability after Synaptic Stimulation

In pyramidal neurons, action potential firing is frequently followed by an afterdepolarization [56,57]. In our experiments, HFS increased the firing frequency and the ADP amplitude in a Ca^2+^_i_-dependent manner and the inhibition of TRPM4 reduced but did not eliminate ADP. The remaining ADP amplitude could be attributed to a combination of channels, particularly T-type Ca^2+^ channels, since the application of Cd^2+^ produced a small reduction in ADP, which is different than what is observed in retrotrapezoid nucleus neurons, where L-type Ca^2+^ channels control the activation of TRPM4 and then, the pacemaker firing [58]. In this respect, our results using Cd^2+^ suggest the participation of a T-type Ca^2+^ channels which are less sensitive to Cd^2+^ than other VGCC, opening the possibility that these channels take part in the activation of TRPM4 in an equivalent way as in thalamic reticular nucleus neurons [54]; however, further experiments need to address this hypothesis. Similarly, HFS still activate an ADP in TRPM4-silenced neurons, showing that the remaining part of the ADP is independent of TRPM4. Notably, 9-Ph, EGTA, and TRPM4 silencing produce similar ADP amplitudes after HFS, strongly suggesting that the residual ADP is independent of Ca^2+^_i_ and TRPM4. In this aspect, several neurons present an ADP dependent in part of a persistent Na^+^ current [59,60,61]; similarly, we hypothesized that this current can be contributing to the Ca^2+^ independent fraction of the ADP observed in our experiments.

A working model for the activation of TRPM4 in pyramidal neurons of the layer 2/3 mPFC after HFS involves the activation of synaptic currents that induce an increase in Ca^2+^_i_ and the induction of post-translational modifications that can increase the channel insertion in the membrane or the change in localization of TRPM4 with respect to the Ca^2+^ source (VGCC or internal stores), resulting in an increase in the TRPM4 current and thus increasing the ADP and the AP firing and changing the excitability of the mPFC layer 2/3 pyramidal neuron. In summary, the results presented here show that TRPM4 plays a key part in the link between synaptic stimulation and intrinsic excitability in a Ca^2+^-dependent way. This interplay between synaptic activity and intrinsic excitability increases the global excitability of the neuron, boosting the probability of neurotransmitter release and improving and/or reinforcing the circuit connectivity. This mechanism of regulation of intrinsic excitability may play a critical role in the physiology of the prefrontal cortex in functions such as working memory and delayed activity.

## 4. Materials and Methods

### 4.1. Animals

All experiments were conducted following the animal protocols approved by the Ethical Committee of the Universidad de Santiago de Chile (#301.2018, 26 May 2018) according to the rules and guidelines of the National Agency of Research and Development (ANID). Briefly, male C57BL/6J mice were housed in a temperature and humidity-controlled facility with a 12/12 h light/dark cycle with water and food ad libitum.

### 4.2. Cell Culture and Transfections

HEK 293T cells were grown in DMEM high glucose supplemented with 10% *v/v* fetal bovine serum and 2 mM glutamine obtained from Thermo Fisher (Carlsbad, CA, USA). Cells were kept at 37 °C and 5% CO_2_ and transfected at 48 h before the experiments using Lipofectamine 2000 according to the manufacturer’s instructions (Carlsbad, CA, USA). Plasmid encoding human TRPM4 FLAG-tagged (pcDNA4/TO-FLAG-hTRPM4) was kindly provided by Dr. P. Launay; plasmid encoding the mouse TRPM4-EGFP (pEGFP-N1-TRPM4b) was kindly provided by Dr. J.M. Simard. 16 to 24 h before the experiment, cells were seeded in 12 mm round coverslips (10^5^ cells).

### 4.3. Stereotaxic Surgery

Layer 2/3 neurons in the mPFC were transduced by injection of lentiviruses containing shRNA for TRPM4 and EGFP (shTRPM4) or the scramble sequence and EGFP (kindly provided by Dr. DJ Linden, John Hopkins University [11,15,62]. C57BL/6J mice (P14 to P20) were anesthetized with isoflurane (3%), the skull was exposed, a small hole was drilled, and the dura was removed. A glass pipette was filled with the lentivirus (5 µL) and connected to a pressure injection device. The viruses (1 µL) were injected at the following coordinates from the bregma (A.P +2.8 mm; M.L. ± 0.2 mm; D.V. −1.2 mm) at a rate of 50 nL/min. All experiments were performed 2 weeks after the injections (P29–P42).

### 4.4. Electrophysiological Recordings

#### 4.4.1. Voltage and Current-Clamp Experiments in Brain Slices

Mice (C57BL/6J) between 4- and 6-week-old were deeply anesthetized with isoflurane (3%) and their brains were quickly removed and placed in ice-cold oxygenated (95% O_2_, 5% CO_2_) high-magnesium ACSF containing (in mM): 124 NaCl, 2.5 KCl, 5 MgCl_2_, 0.5 CaCl_2_, 1.25 NaH_2_PO_4_, 25 NaHCO_3_, 11 Glucose, and pH 7.4. Tissue blocks containing the prefrontal cortex were cut using a vibratome to obtain coronal brain slices (350 µm thick). Then, slices were transferred to a chamber with oxygenated ACSF containing (in mM): 125 NaCl, 2.5 KCl, 1.3 MgCl_2_, 2.5 CaCl_2_, 1.25 NaH_2_PO_4_, 25 NaHCO_3_, 11 Glucose, and pH 7.4. After 1.5 h recovery, the slices were transferred to a recording chamber mounted on a Zeiss Axio Examiner A1 DIC microscope (Oberkochen, Germany). Slices were continuously perfused with oxygenated ACSF (2–3 mL/min) at 34 ± 2 °C.

Whole-cell recordings were performed from layer 2/3 pyramidal neurons in the mPFC using borosilicate glass pipettes (4 to 6 MΩ). For current clamp recordings, intracellular solution contained (in mM): 130 potassium gluconate, 10 KCl, 10 HEPES, 0.5 EGTA (unless otherwise indicated), 2 Mg-ATP, 0.3 Na-GTP, pH 7.2 adjusted with KOH (~300 mOsm, liquid junction potential (LJP) = 14.9 mV); and for voltage-clamp recordings the intracellular solution contained (in mM): 120 CsCh_3_SO_3_, 10 CsCl, 10 HEPES, 5 TEA-Cl, 0.5 EGTA, 2 Mg-ATP, 0.3 Na-GTP, 5 QX-314, and pH 7.2 adjusted with CsOH (~300 mOsm, LJP = 8.4 mV). For the perforated patch protocol, 300 µg/mL nystatin (which permeates only monovalent ion [63]) diluted in DMSO and 1.5 mM EGTA (2 mM final, pH 7.2) were added to the intracellular solutions. For perforated patch recording, a stable access resistance (R_a_) was obtained after 5 to 10 min (R_a_ = 10–20 MΩ) [64]. When a sudden decrease in the access resistance occurred, the recording was discarded. All salts and reagents were obtained from Sigma-Aldrich (Burlington, MA, USA).

To measure the input-output function, a depolarizing current step protocol was applied (1 s, −200 to 800 pA, 100 pA step) with a holding current of 0 pA. The EPSP measurements were performed in the presence of 100 µM Picrotoxin. To test the excitability after synaptic stimulation, a somatic current injection of 400 pA was used. Current-clamp recordings were bridge-balanced, and R_s_ was monitored during the experiment using a somatic hyperpolarizing current pulse (−50 pA, 50 ms). Cells showing changes > 20% in R_s_ were discarded from the analysis. To measure the ADP, a somatic suprathreshold depolarizing current (2 nA) was injected for 2 ms into the soma.

For voltage-clamp recordings, pipettes, whole-cell capacitance, and series resistance were compensated by >80%. Neurons were held at −70 mV and recorded in the ACSF with a cocktail of inhibitors (CI) containing (in µM): 1 TTx, 50 CNQx, 25 DL-AP5, 100 CdCl_2_, and 100 Picrotoxin to isolate the TRPM4 current. Electrophysiological recordings were performed using a Multiclamp 700A (Molecular Devices, San Jose, CA, USA) or HEKA EPC10 (HEKA GmbH, Reutlingen, Germany) data was filtered at 10 kHz and digitized at 20 kHz using pClamp 10.3 or HEKA Patchmaster 2.91. For the extracellular high frequency stimulation protocol (HFS), a tungsten bipolar electrode (FHC) was placed at 200 µm away from the soma in the mPFC layer 1, and 10 trains repeated at 0.3 Hz, with each train composed of 10 stimulation pulses at 100 Hz, and using a stimulus intensity of 50% of the maximal slope (200–300 µA, pulse of 50 µs), delivered using an A365 stimulus isolator (WPI, Sarasota, FL, USA). For the experiments of TRPM4 pharmacological modulation, we used 10 µM 9-Phenanthrol (9-Ph) (Sigma-Aldrich, Burlington, MA, USA) or 100 nM BTP-2 (Tocris, Minneapolis, MN, USA) perfused to the whole brain slice.

#### 4.4.2. TRPM4 Current Measurements in Overexpression System

Voltage-clamp recordings were performed in HEK293T cells expressing the murine or human TRPM4 channel. The extracellular solution contained (in mM): 140 NaCl, 5 KCl, 2.5 CaCl_2_, 1 MgCl_2_, 10 HEPES, 10 Glucose, and pH 7.4. The intracellular solution was (in mM): 140 CsCl, 5 NaCl, 1 MgCl_2_, 10 HEPES, and pH 7.2 adjusted with CsOH (LJP = 3.8). Ca^2+^ concentrations were adjusted between 0.01 to 10 µM of free calcium by adding the total amounts of CaCl_2_ and 1 mM of Ca^2+^ chelator (HEDTA, EGTA, BAPTA, or citric acid) as calculated by WebMaxc (https://somapp.ucdmc.ucdavis.edu/pharmacology/bers/maxchelator/webmaxc/webmaxcS.htm, accessed on 21 February 2020) (Supplementary Figure S1). Voltage clamp experiments were performed in Axopatch 200B and HEKA EPC10 and Digidata 1440 (Molecular Devices, San Jose, CA, USA), data were filtered at 5 kHz and digitized at 10 kHz. Voltage step protocols were delivered at 0.2 Hz and consisted of a hyperpolarizing step of −80 mV (500 ms) followed by a depolarizing step at 80 mV (500 ms), from a holding potential of 0 mV. Voltage was not corrected for liquid junction potential and all experiments were performed at 22 ± 2 °C.

### 4.5. Data Analysis

Electrophysiological data were analyzed using Clampfit 10.3 (Molecular Devices, San Jose, CA, USA) and Igorpro 6.37 (Wavemetrics, Lake Oswego, OR, USA) with Neuromatic module [65]. Rheobase was defined as the minimal current injection needed to elicit an AP. The doublet firing at the beginning of the step was defined as the time between the first two APs at 400 pA somatic current injection. Data are reported as mean ± SD unless stated otherwise. We tested the data for normal distribution using the Kolmogorov–Smirnov test and the statistical significance between group means was evaluated by one-way ANOVA followed by Dunnett’s multiple comparison post hoc test using GraphPad Prism 8 (San Diego, CA, USA). In some cases, *t*-tests were used. For non-parametric data, we used the Kruskal–Wallis test. Statistical significance was determined at *p* < 0.05. For effect size analysis, we used mean difference calculations, and the statistical significance of the mean difference is presented above each Gardner–Altman estimation plot and was calculated utilizing a two-side permutation *t*-test using Estimation Statistic beta (http://www.estimationstats.com, accessed on 10 March 2021).

## Data Availability

All data reported in this work are available upon request.

## Supplementary Materials

The following are available online at https://www.mdpi.com/article/10.3390/ijms22105268/s1. Figure S1: Ca^2+^ sensitivity of TRPM4 in overexpression systems, Figure S2: Effect of shTRPM4 in the levels of TRPM4.
