# Supplementary material for: ICAN (TRPM4) Contributes to the Intrinsic Excitability of Prefrontal Cortex Layer 2/3 Pyramidal Neurons
Source: Int J Mol Sci. 2021 May 17;22(10):5268. doi: 10.3390/ijms22105268 (PMC8157065; doi:10.3390/ijms22105268)
Supplement: Supplementary file 1 [file ijms-22-05268-s001.zip › ijms-1213093-supplementary.pdf]

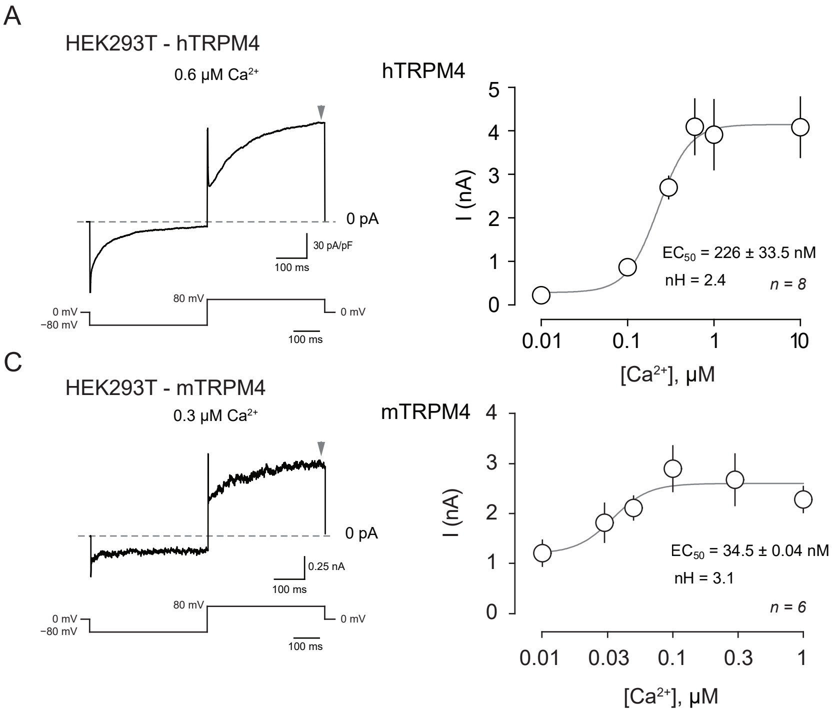

B

hTRPM4	Chelator	Free Ca^{2+} (μM)	Total Ca^{2+} (μM)
	EGTA	0.01	61
	EGTA	0.1	393
	BAPTA	0.3	701
	BAPTA	0.6	824
	Citrate	1	42
	HEDTA	10	685

mTRPM4	Chelator	Free Ca^{2+} (μM)	Total Ca^{2+} (μM)
	BAPTA	0.01	72.4
	BAPTA	0.03	189.9
	BAPTA	0.05	281
	BAPTA	0.1	438.7
	BAPTA	0.3	701.2
	BAPTA	1	887.5
	EGTA	0.1	393.9

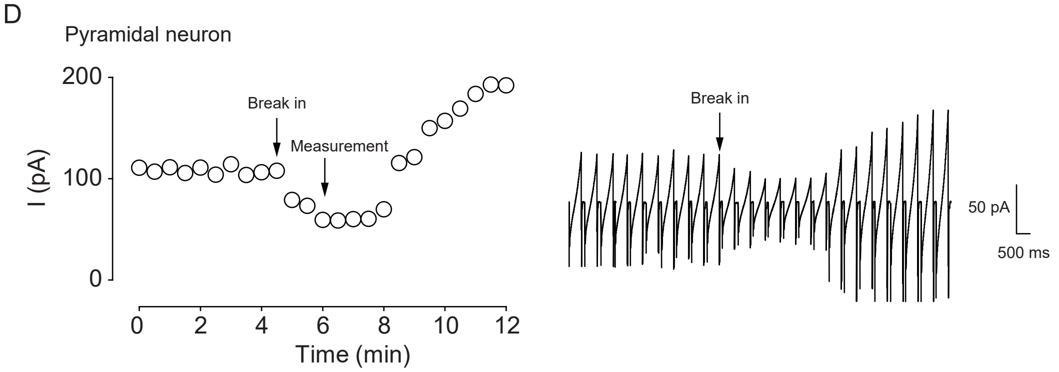

Supplementary Figure S1

Ca^{2+} sensitivity of TRPM4 in overexpression systems. HEK 293T cells were transfected with hTRPM4 or mTRPM4. After the 24 h expression, TRPM4 current was evaluated at several calcium concentrations. The current values were plotted as a function of the concentration and a Hill equation was fit to the results. (A) Shows the effect of Ca^{2+} on hTRPM4. (B) Show the different concentrations of free Ca^{2+}, total Ca^{2+}, and the Ca^{2+} chelator. (C) Shows the effect of mTRPM4. In both cases, the right panels show the representative current at 0.6 μM (hTRPM4) or 0.3 μM (mTRPM4), the voltage step protocol it is showed below the current trace. The arrows indicate where the current was measured. (D) Shows the time course of the current measured in pyramidal neurons in layer 2/3 of the mPFC in response to a voltage ramps using the nystatin perforated patch technique, the black arrow indicate the break in the membrane patch to enter in whole cell allowing the difussion of EGTA, the grey arrow indicate the area where the measurements were taken, currents were measure at 80 mV.

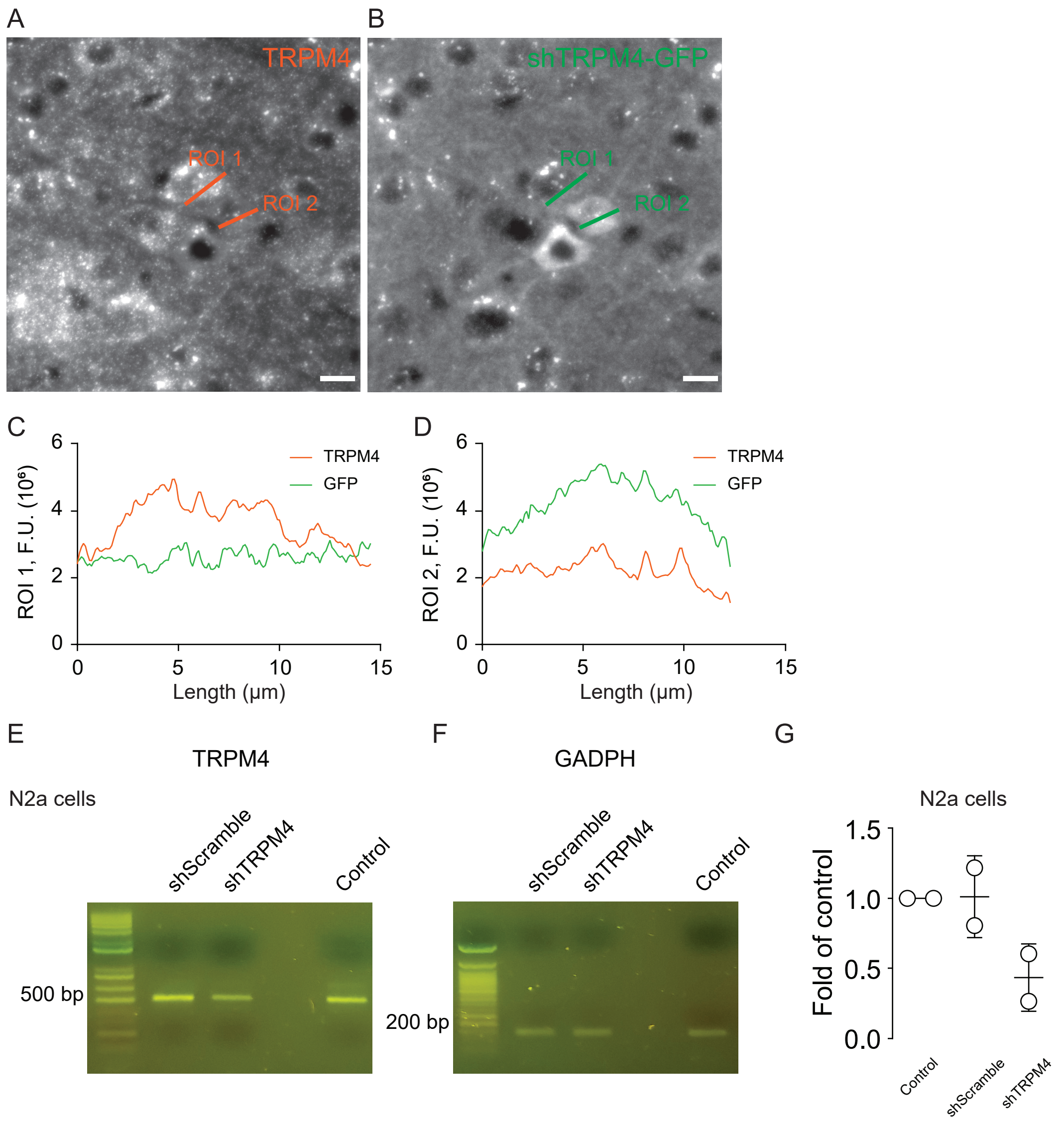

Supplementary Figure S2

Effect of shTRPM4 in the levels of TRPM4. Confocal image showing pyramidal neurons in the L2/3 of the mPFC expressing shTRPM4-GFP and stained with anti-TRPM4 antibody. (A) Shows the labeling for TRPM4. (B) Show the GFP signal, the lines indicate the region used for the analysis. C and D shows the fluorescence intensity of TRPM4 labeling and GFP in non-transduced (C) and transduced neurons (D). RT-PCR from N2a neurons expressing scramble, shTRPM4 and non-transduced cells. (E) Show TRPM4 amplification and in (F) GADPH amplification. (G) Quantification of the product of amplification of TRPM4, data were normalized to the expression of the GADPH housekeeping gene and expressed as a fold of the control (non-transduced cells).
